# A Popular View of Homœopathy

**Published:** 1835-01-01

**Authors:** 


*A Popular View of Homœopathy.* By the Rev. Thomas Everest, Rector of Wickwar, Gloucestershire.—London, 1834. Small 8vo. pp. 95.

This is a very clever little treatise, sparkling, pleasant, readable; yet does its author fall into three enormous mistakes. The first is, in supposing that medical men wish to persecute him and his hobby; or, to use a common phrase, that the doctors want to make a dead set at him. The second is, in imagining that very little progress has been made in modern times in the practice of physic. Our author's third mistake (the most astounding and incomprehensible of the three,) consists in the supposition that physicians are averse to novelties; that they are quite satisfied with the art of healing as it stands, and reject any attempt to improve upon the orthodox treatment, as a schoolman of the middle ages would have scouted the would-be refuter of Aristotle. This is not a mere slip of the good rector's pen, for it occurs more than once. Thus, at p. 20, he says, "However, this may be denied by those on whom education and interest have combined to impose the belief that human intellects are incapable of admitting any more knowledge on the subject of medicine than has been already revealed to the members of the College of Physicians," &c. Again, at p. 37 he copies Hahnemann, and declares that

"First of all, certain general therapeutic qualities are attributed to particular substances. One is still, as it was said to be in the time of Dioscorides, seventeen hundred years ago, a diuretic; another a sudorific; a third an anodyne; a fourth an anti-spasmodic, &c. And supposing all this to be literally true, (which he asserts that it very rarely is when brought to the test of experiment,) what has been learnt excepting one property of each medicament? Nothing whatever is known of the action of any one on the rest of the organism; nothing of its special and peculiar power of affecting the rest of the frame; nothing of its influence and action on that part of the organism which is already affected, or on that which is not affected. Taken in large quantities, such and such a substance for instance is an anodyne. Thus much is supposed to be known, and it is prescribed and taken accordingly. A very desirable end, no doubt, to relieve pain! But who knows what symptoms of other kinds it is exciting all the while? Who knows what suffering it is preparing in return for the pain it has temporarily extinguished? If new symptoms follow the use of it, they are referred to the disease perhaps, or perhaps looked on as imaginary, and the patient declared to be affected with that curious complaint which baffles medicine, hypochondria!"

And then, at p. 80, he treasonably insinuates that doctors do not read Journals. Hear him!

"If such a man, so loaded with onerous duties, with such grave responsibilities, and such solemn consequences depending on him, were well aware of the nature of his office, he would shrink from the notion that he had already exhausted all information on the subject of healing; he would doubt his own powers; he would distrust his own judgment; he would weary Heaven with prayers for light and knowledge; he would deem it criminal to waste in indolent acquiescence an hour that might have been employed in investigation, and a sin against the Majesty of heaven to reject one single assertion connected with the science of healing, until he had convinced himself by actual trial of its falsehood. The four winds would bring him tidings of all new discovered simples; and not a finger-ach could be cured above the line of perpetual snow, but, like Fine-ear, in the tale, he would catch the whisper of it along the earth." (P. 80.)

Now we affirm that doctors read Journals most diligently, and consequently obtain tidings of all new-discovered simples, at a much smaller expense than that of keeping Eurus and Boreas in pay: yes, and these useful repositories may be considered in the light of acoustic tubes, conducting the faint sounds of distant knowledge into the auditory meatus of persons who have no pretensions to be *Fine-ears.* Surely, Mr. Everest (who evidently takes in the Medical Gazette,) cannot open a number of our excellent contemporary, without seeing some new discovery in the art of healing; but he will very rarely indeed find any attempt to crush the novelty by the mere weight of authority.

We shall not give an analysis of Mr. Everest's work, as we have so frequently detailed the merits and demerits of Hahnemann's theory, and gave a long account of his Organon, in our first Number, but shall content ourselves with one more quotation. The following observations on what might be called *druggery,* by which a human stomach is turned into an ill-assorted apothecary's shop, are far from bad.

" How much of the disease at present existing in the world has been produced by natural morbific causes, and how much resulted from the improper use of medicine, it might be difficult to determine. We cannot err, however, in asserting that every grain of medicine taken beyond what was necessary to effect a cure, has been productive of suffering, for which they who prescribed it are alone responsible. When we recollect the wasting deluge of drugs with which the uncertainty of the art of medicine has enabled every wretched empiric to flood this country, the pocket-book recipes, grandmother nostrums, family receipts, domestic formulæ, patent medicines, family medicine chests, and other perennial and inexhaustible fountains of mischief,—the multitudinous compounds of modern pharmacy, the random mixtures, the multiplied bottles and boxes which, unlike Pandora's, have not even hope at the bottom, and the utter recklessness in drug-swallowing, which has been confirmed, if not originally introduced, by the colossal doses prescribed by physicians, we cannot but believe that much of the suffering to be found in this island is due to the abuse of those very substances which a kind Creator has provided for man's restoration." (P. 34.)

Appendix I. contains two cases cured homœopathically.

A. B. (apparently our good rector,) was suffering under dyspepsia of long standing, abscesses on the toe and thumb, a whitlow on the finger, a spreading ulcer on the chin, and another on the ear. He began taking his decillionths of belladonna on the 8th of December, and was cured by the 3d of January.

A child, aged two years, who had been afflicted from her birth with very obstinate constipation, was cured in a few weeks by homœopathic treatment. It is not stated what were the remedies used.

We would now give our worthy author two serious pieces of advice. First, let him send his homœopathic benefactor, who healed his ulcerated ear, &c., or some of his disciples, into our London hospitals, and let them cure half a dozen bad cases in the presence of the sages of physic: this will do the Meissen doctrine far more good than a thousand exhortations to the incredulous to put it into practice. Secondly, let Mr. Everest bring up all his sons, nephews, and younger brothers, to homœopathy; for, if one half of what he says be true, some ten years hence homœopathy will be considered to bear the same relation to allopathy that a case-full of Lucifer matches does to a tinderbox; homœopathy being a cheap and easy method of diffusing the light of health, and allopathy being too apt to seduce the operator into giving himself very severe raps on the knuckles.

